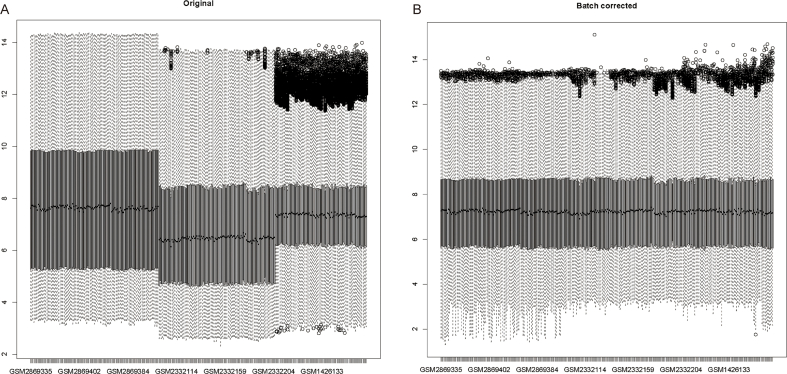# Comprehensive evaluation of disulfidptosis in intestinal immunity and biologic therapy response in Ulcerative Colitis

**DOI:** 10.1016/j.heliyon.2024.e34516

**Published:** 2024-07-19

**Authors:** Lichao Yang, Lianwen Yuan, Ganglei Liu

**Affiliations:** Department of General Surgery, The Second Xiangya Hospital of Central South University, 410011, Changsha, China

**Keywords:** Ulcerative Colitis, Disulfidptosis, GEO dataset, Biologics, Immune, Inflammatory bowel disease

## Abstract

**Objective:**

Ulcerative Colitis (UC) manifests as a chronic inflammatory condition of the intestines, marked by ongoing immune system dysregulation. Disulfidptosis, a newly identified cell death mechanism, is intimately linked to the onset and advancement of inflammation. However, the role of disulfidptosis in UC remains unclear.

**Methods:**

We screened differentially expressed genes (DEGs) associated with disulfidptosis in multiple UC datasets, narrowed down the target gene number using lasso regression, and conducted immune infiltration analysis and constructed a clinical diagnostic model. Additionally, we explored the association between disulfidptosis-related key genes and disease remission in UC patients receiving biologic therapy. Finally, we confirmed the expression of key genes in FHC cells and UC tissue samples.

**Results:**

In the differential analysis, we identified 20 DEGs associated with disulfidptosis. Immune infiltration results revealed that five genes (PDLIM1, SLC7A11, MYH10, NUBPL, OXSM) exhibited strong correlations with immune cells and pathways. Using GO, KEGG and WGCNA analyses, we discovered that gene modules highly correlated with disulfidptosis-related gene expression were significantly enriched in inflammation-related pathways. Additionally, we developed a nomogram based on these five immune-related disulfidptosis genes for UC diagnosis, showing robust diagnostic capability and clinical efficacy. Kaplan-Meier survival analysis revealed a significant link between changes in the expression levels of these cell genes and disease remission in UC patients receiving biologic therapy. In line with previous studies, similar expression changes of the target gene were seen in both UC cell models and tissue samples.

**Conclusions:**

This study utilized bioinformatic analysis and machine learning to identify and analyze features associated with disulfidptosis in multiple UC datasets. This enhances our comprehension of the role disulfidptosis plays in intestinal immunity and inflammation in UC, providing new perspectives for developing innovative treatments for UC.

## 1 Introduction

Ulcerative Colitis (UC), a predominant form of inflammatory bowel disease (IBD) affecting the colon and rectum, is characterized by recurrent and incurable features, clinically presenting as mucopurulent bloody stools and persistent intestinal inflammation [1]. The etiology of UC remains unclear, likely involving factors such as genetics, environment, lifestyle, gut microbiota, and immune dysregulation [2]. In recent years, the incidence of UC worldwide has steadily increased alongside improving living standards, placing a considerable strain on the global economy and healthcare systems [3]. Treatment of UC often involves medications like 5-aminosalicylic acid, steroids, immunosuppressants, and biologics. However, extended use of these drugs can result in resistance and additional side effects [4]. Moreover, UC often faces challenges in clinical diagnosis, potentially resulting in delayed systemic treatment and affecting the efficacy of later-stage interventions for patients [1,5]. Therefore, elucidating the pathogenesis of UC is crucial for discovering new therapeutic targets and diagnostic markers, significantly enhancing clinical symptom relief for UC patients and improving the efficiency of UC drug treatments.

As an immune-imbalanced disease, cell death plays a crucial role in the pathogenesis and progression of UC, essential for maintaining stable intestinal environments and regulating gut ecology [6]. Disulfidptosis, a recently discovered cell death mechanism induced by disulfide bond stress, represents a novel mechanism closely associated with inflammation initiation and progression. An abnormal accumulation of intracellular disulfides, such as cysteine, induces disulfide stress, which is highly toxic to cells and triggers cell death [7,8]. Nicotinamide adenine dinucleotide phosphate (NADPH), as a reducing agent, can reduce disulfides and prevent cell damage [7]. Disulfidptosis is considered to play a crucial role in cancer metabolism therapy [9]. The accumulation of dead cells is likely to activate immune cells, induce inflammatory responses, and potentially impact local metabolism [10]. However, in the immune dysregulation of UC, there is recurrent intestinal inflammation leading to disrupted gut metabolism. Whether disulfidptosis participates in the inflammation within the UC intestine is not yet clear. Therefore, this study aims to explore the role of disulfidptosis in UC intestinal immunity and inflammation, providing further theoretical evidence for how cell death influences the disease progression of UC.

**Fig. 1:**
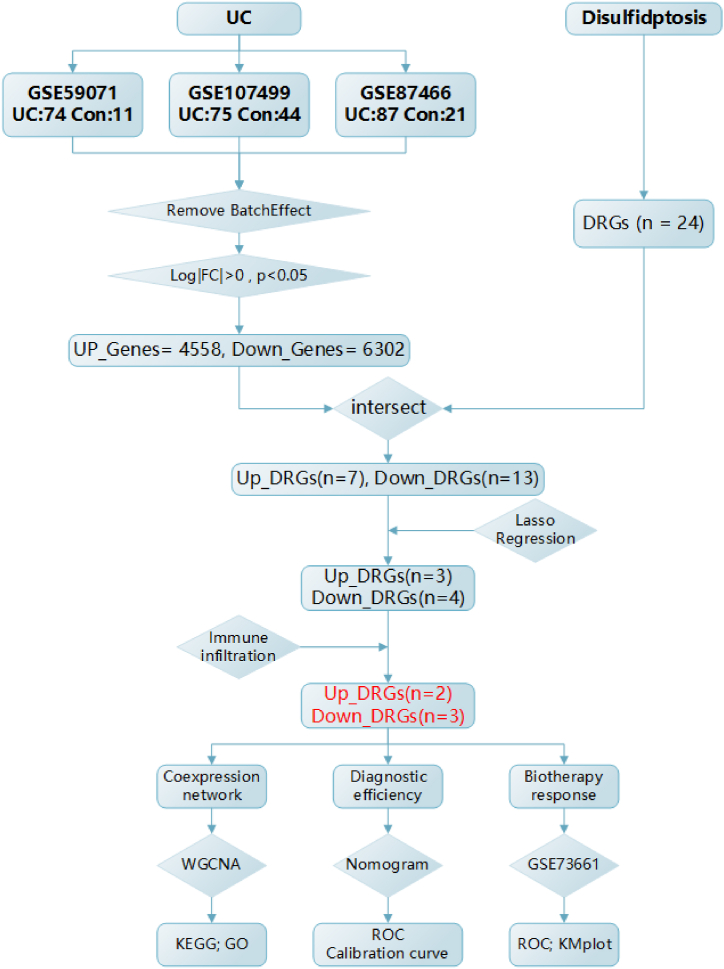
The flow chart of the study, including experimental grouping design and research process. (UC: Ulcerative colitis; DRGs: Disulfidptosis Related Genes).

In this study, we downloaded three datasets (GSE107499, GSE87466, GSE59071) containing gene expression data from UC mucosal tissue in the GEO database. After merging and screening for differentially expressed genes (log|FC| > 0, p-value <0.05), we intersected these genes with disulfidptosis-related genes, resulting in 20 differentially expressed genes. Subsequently, we used lasso regression to further filter the genes obtained in the previous step, ultimately identifying seven differentially expressed disulfidptosis-related genes (Fig. 1). Additionally, we observed the immune infiltration landscape within UC intestines, selecting five immune-related disulfidptosis genes based on their correlation with immune cells or pathways (up = 2, down = 3). To further explore their diagnostic capabilities in UC, we constructed a nomogram using these five key genes for diagnosing UC and evaluated the clinical efficacy of this diagnostic model. Finally, we downloaded dataset GSE73661 to validate the performance of these five genes in UC biologic therapy (vedolizumab (VDZ) and infliximab (IFX)).

## 2 Materials and methods

### 2.1 Data sources

We searched the GEO database for UC-related datasets and selected GSE107499, GSE87466, GSE59071, and GSE73661 for analysis. A total of 312 colon mucosal tissue samples were included, comprising 236 samples from UC patients in the active disease phase and 76 samples from normal colon mucosa. We integrated the gene expression data from GSE107499, GSE87466, and GSE59071, and eliminated batch effects from the resulting expression matrix for differential analysis. After identifying the target genes, we used the GSE73661 dataset to evaluate the influence of VDZ and IFX on these genes' expression. This enabled us to determine the effectiveness of pivotal genes in predicting whether UC patients achieve disease remission following biologic therapy.

### 2.2 Identification of differentially expressed genes associated with disulfidptosis

We collected 24 genes related to disulfidptosis through the literature review for subsequent analysis [8,11,12]. In order to further narrow down the selection of target genes, we identified differentially expressed genes in the merged UC colon mucosal expression matrix using the criteria of fold change (log|FC|) > 0 and p < 0.05. Subsequently, we intersected the differentially expressed genes with those associated with disulfidptosis, resulting in a total of 20 genes. To illustrate these findings, we generated a Venn diagram.

### 2.3 Lasso regression

We applied Lasso regression to sift through the 20 differentially expressed genes identified in the preceding step. Employing a 10-fold cross-validation approach for iterative analysis, we derived a model exhibiting outstanding performance while utilizing the fewest variables. The outcome revealed 7 pivotal genes, encompassing 3 upregulated genes and 4 downregulated genes.

### 2.4 Analyses of immune infiltration and immune correlation

To increase the reliability of the immune infiltration results, we utilized both the “CIBERSORT” and “ssGSEA” algorithms for analyzing the UC dataset [13,14]. We conducted correlation analyses on the previously selected 7 differentially expressed genes associated with disulfidptosis, using a significance threshold of p < 0.05. Based on the criteria of |(Ps)| ≥ 0.50 in both CIBERSORT and ssGSEA results, we ultimately identified 5 genes associated with disulfidptosis that showed significant correlation with immune dysregulation in the UC intestinal context. Subsequent analysis involved WGCNA (Weighted Gene Co-expression Network Analysis).

### 2.5 WGCNA analysis

To further investigate the intrinsic connections among disulfidptosis-related genes significantly correlated with immune dysregulation in the UC intestinal context, we employed Weighted Gene Co-expression Network Analysis (WGCNA). This method facilitated the construction of a gene co-expression network to identify gene modules exhibiting co-expression patterns with the 5 identified genes [15,16].

### 2.6 Functional annotation and pathway enrichment analysis

In order to further elucidate the role of differentially expressed genes in biological processes, we conducted Gene Ontology (GO) and Kyoto Encyclopedia of Genes and Genomes (KEGG) analyses [17]. To ensure the reliability of the results, we utilized a significance threshold of p < 0.05 for the identification of significantly enriched GO terms and KEGG pathways.

### 2.7 Diagnostic model

The selected 5 key genes were used to construct a diagnostic model for UC (Nomogram), and the expression matrix was randomly divided into training and validation sets in a 7:3 ratio. In the training set, we applied an algorithm to establish a clinical prediction nomogram. To further validate its accuracy, we subjected the constructed model to examination using a validation set. Finally, we plotted a calibration curve to assess the calibration performance of the nomogram prediction model.

### 2.8 Validation of the disulfidptosis related genes signature

To validate the value of the target genes in the diagnosis of UC patients, we conducted ROC analysis to assess their predictive capability. We quantified the area under the ROC curve (AUC value) for each gene. Additionally, we employed unsupervised consensus clustering to group all UC patients and performed Kaplan-Meier (KM) survival analysis on different patient clusters [18].

### 2.9 Cell culture and treatment

The human normal colonic epithelial cell line (FHC) used in this study was purchased from the American Type Culture Collection (ATCC, Rockville, Maryland), and cultured in DMEM high glucose medium supplemented with 10 % fetal bovine serum and 1 % penicillin/streptomycin. Cells were maintained in a humidified atmosphere with 5 % CO_2_ at 37 °C and observed under a microscope every 24 h. To simulate the cellular environment of UC, FHC cells were seeded in 6-well plates and treated with 50 ng/ml LPS solution when the cell density reached an appropriate level. After 12 h, cells were harvested for RNA or protein extraction for subsequent experiments.

### 2.10 Collection of UC mucosal tissue specimens

The four IBD clinical intestinal lesion tissue samples in this study were obtained from UC patients who underwent surgery at the Second Xiangya Hospital of Central South University from December 2022 to December 2023 (all patients were pathologically diagnosed with UC, including 2 males and 2 females). Relative normal intestinal control samples were taken from intestinal tissues approximately 5 cm away from the ulcer (confirmed by pathological examination to be normal intestinal mucosa). All tissue specimens were collected within 30 min of bowel specimen excision and stored directly in liquid nitrogen for subsequent research. Informed consent was obtained from all patients participating in this scientific study, and the study was approved by the Ethics Committee of the Second Xiangya Hospital of Central South University (Approval No: 2022-155).

### 2.11 qRT-PCR

RNA was extracted from cells or intestinal mucosal tissues using TRIZOL reagent, reverse transcribed, and used to detect the mRNA expression levels of target genes. The primers used in this study are listed in Supplementary Table 2.

### 2.12 Statistical analysis

All data processing and statistical analyses were performed using R software (version 4.2.0), and the executable code can be found in the supplementary files. All figures were generated using Adobe Illustrator software (version 2022). A significance level of p < 0.05 was considered statistically significant, denoted as (*p < 0.05, **p < 0.01, ***p < 0.001).

## 3 Result

### 3.1 Identification of disulfidptosis related DEGs

**Fig. 2:**
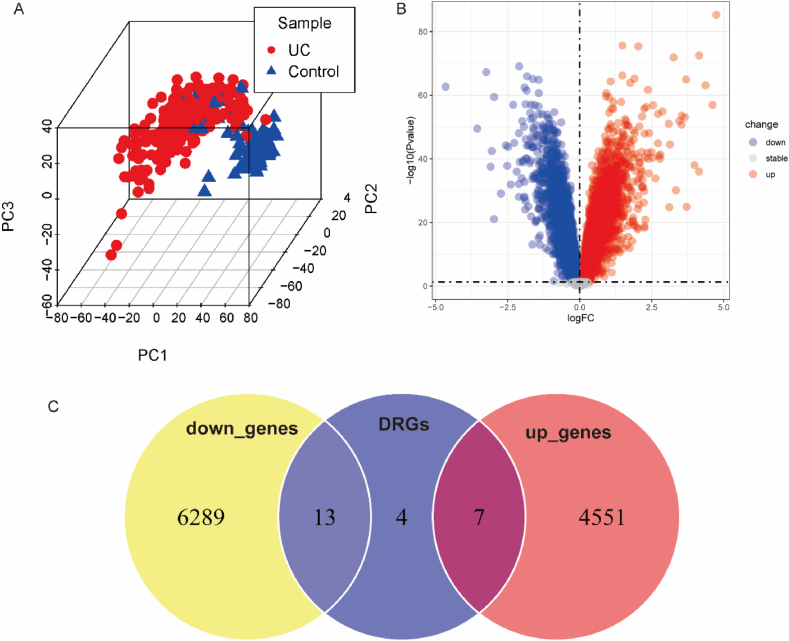
These 3 datasets GSE107499, GSE87466 and GSE59071 were merged and normalized to a new dataset. A. The Principal Component Analysis of the new dataset. B. Volcano plot of DEGs, the red nodes represent the 4558 significantly upregulated DEGs and the blue nodes show the 6302 downregulated DEGs based on |log2 FC|> 0 and p < 0.05. C. Intersection of DEGs and disulfidptosis related genes. (UC: Ulcerative colitis; DEGs, differentially expressed genes; DRGs: Disulfidptosis Related Genes). (For interpretation of the references to color in this figure legend, the reader is referred to the Web version of this article.)

**Fig. 3:**
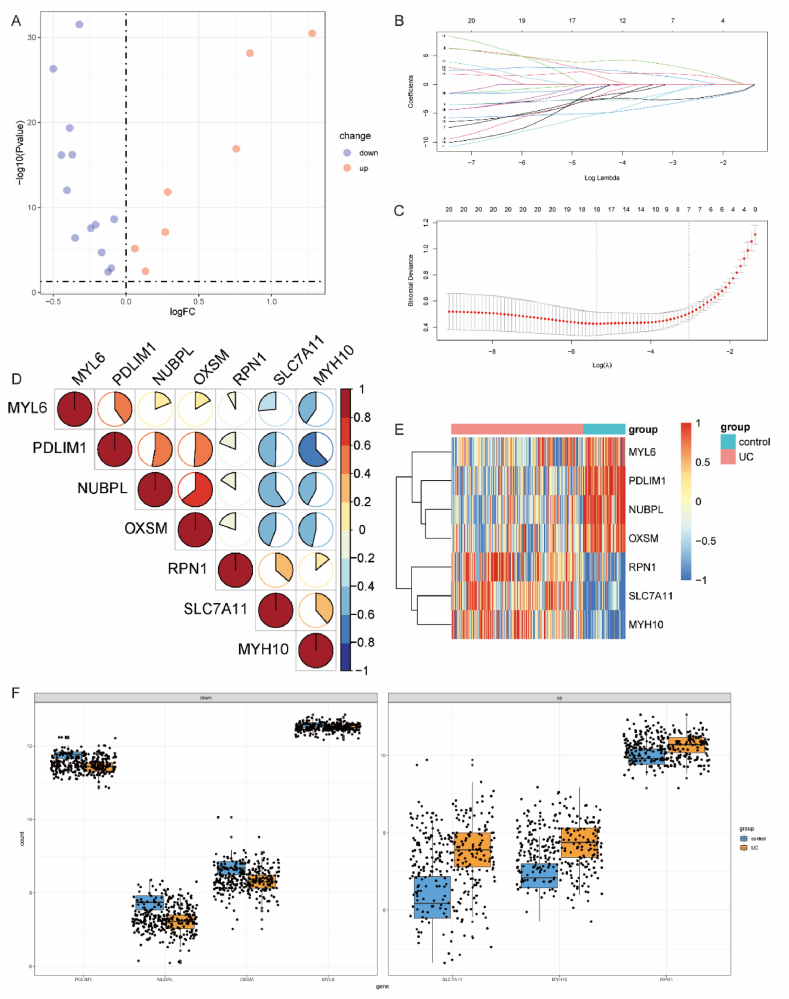
Further selection of differentially expressed genes associated with disulfidptosis. A. Volcano plot illustrating the expression of differentially expressed genes related to disulfidptosis in the merged dataset (|log2 FC| > 0 and p < 0.05). B. Distribution plot of LASSO coefficients for genes associated with disulfidptosis. C. Cross-validation plot of penalty terms, indicating that 18 variables were retained when the error was minimized, corresponding to the position indicated by the left dashed line. To avoid overfitting and simplify the model, compared to the minimum error, the selected standard error did not exceed 1, retaining 7 variables, corresponding to the position indicated by the right dashed line. D. Correlation heatmap of the six key genes. E. Heatmap displaying the expression of these six key genes in the merged dataset. F. Boxplot showing the expression levels of target genes between UC and the normal control group.

First, we merged the three UC datasets and removed batch effects (Supplementary Figures 1A and B). The 3D PCA plot displayed distinct clustering of UC and control groups (Fig. 2A). Applying the criteria of log|FC|>0 and p < 0.05, we identified 4558 upregulated genes and 6302 downregulated genes (Fig. 2B). Next, the intersection of 24 disulfidptosis-related genes from literature review (Supplementary Table 1) and differentially expressed genes yielded 20 genes, comprising 7 upregulated and 13 downregulated genes (Fig. 2C). The volcano plot illustrated the expression profile of these genes in the dataset (Fig. 3A). Further target gene selection was performed through Lasso regression on the expression data of these 20 genes, resulting in a model with excellent performance and minimal variables, identifying 7 key genes (Fig. 3B and C). The heatmap displayed the expression differences of these 7 genes in UC (Fig. 3E), correlation analysis indicated internal associations among these 7 differentially expressed genes (Fig. 3D), and the boxplot depicted the expression level variations of target genes between UC and normal control groups (Fig. 3F).

The Immune Infiltration Profile in UC and the Identification of Immune-Related Genes Associated with Disulfidptosis.

**Fig. 4:**
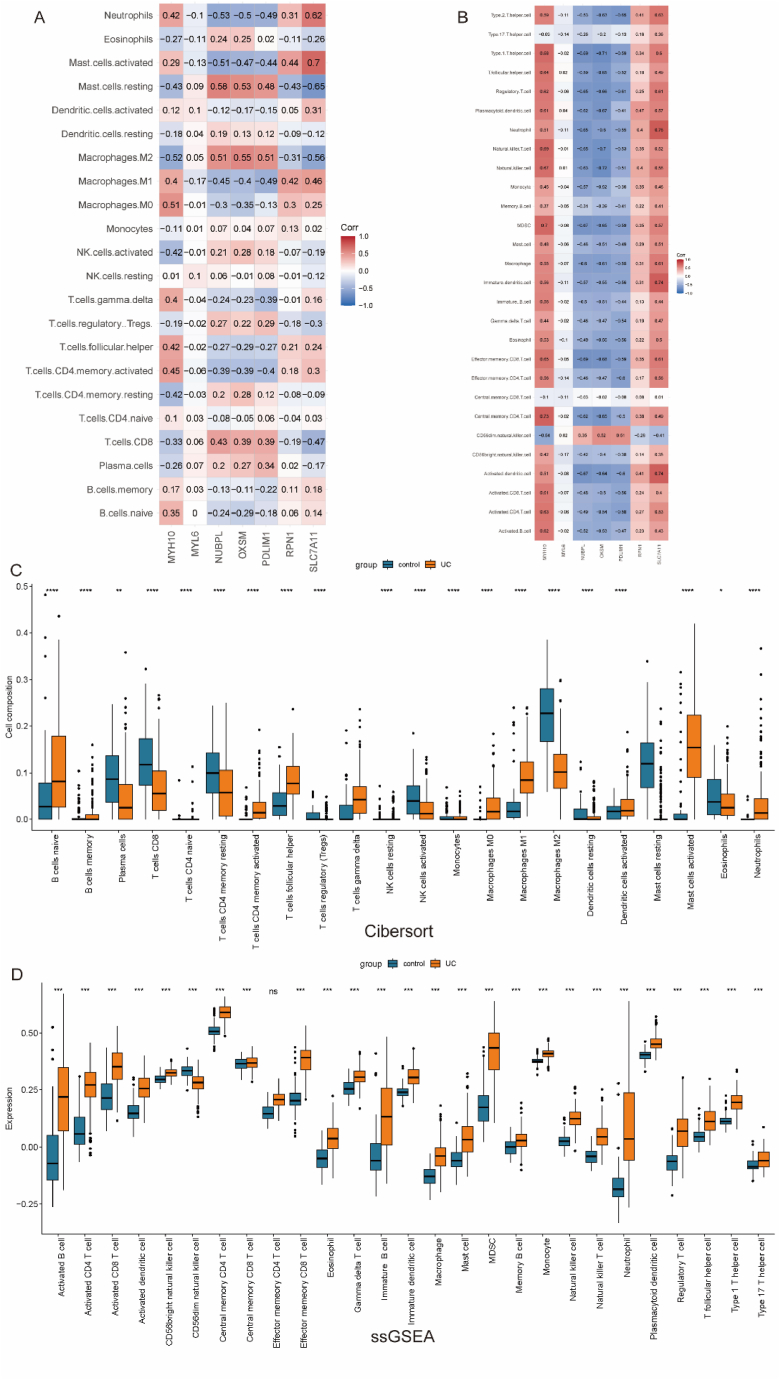
Immune infiltration landscape in UC. A. Correlation matrix of enrichment scores for disulfidptosis-related DEGs calculated by CIBERSORT. B. Correlation matrix of enrichment scores for disulfidptosis-related DEGs calculated by ssGSEA. C. Comparison of immune cell infiltration between the UC group and the normal control group calculated by CIBERSORT. D. Comparison of immune cell infiltration between the UC group and the normal control group calculated by ssGSEA. (*p < 0.05, **p < 0.01, ***p < 0.001).

Cell death is associated with immune dysregulation, but the connection between changes in the immune microenvironment in the UC intestine and disulfidptosis remains unclear. To gain deeper insights into the pathogenesis of UC, we employed two different algorithms, CIBERSORT and ssGSEA, to compare immune cell infiltration levels in the intestinal mucosa of UC patients. Specifically, CIBERSORT analysis revealed T cell activation in both UC and the normal control group, while the infiltration level of NK cells in UC patients was significantly lower than in the control group. Additionally, there was a significant increase in M1-type pro-inflammatory macrophages and neutrophil infiltration in UC (Fig. 4C). Similar results were observed in the ssGSEA analysis (Fig. 4D), indicating a potential excessive immune response in the UC intestine.

To further elucidate the connection between disulfidptosis and the occurrence and development of UC, we conducted a correlation analysis between the 7 key genes selected in the previous step and the abundance of immune cells. As expected, these 7 key genes showed a strong correlation with immune cells (Fig. 4A and B), suggesting a close association between disulfidptosis and UC. Using correlation coefficients (0.50) and p-values derived from both algorithms, we identified 5 genes (PDLIM1, SLC7A11, MYH10, NUBPL, OXSM) showing the strongest correlation for subsequent analysis.

### 3.2 Construction and functional annotation of key modules for immune-related disulfidptosis DEGs

**Fig. 5:**
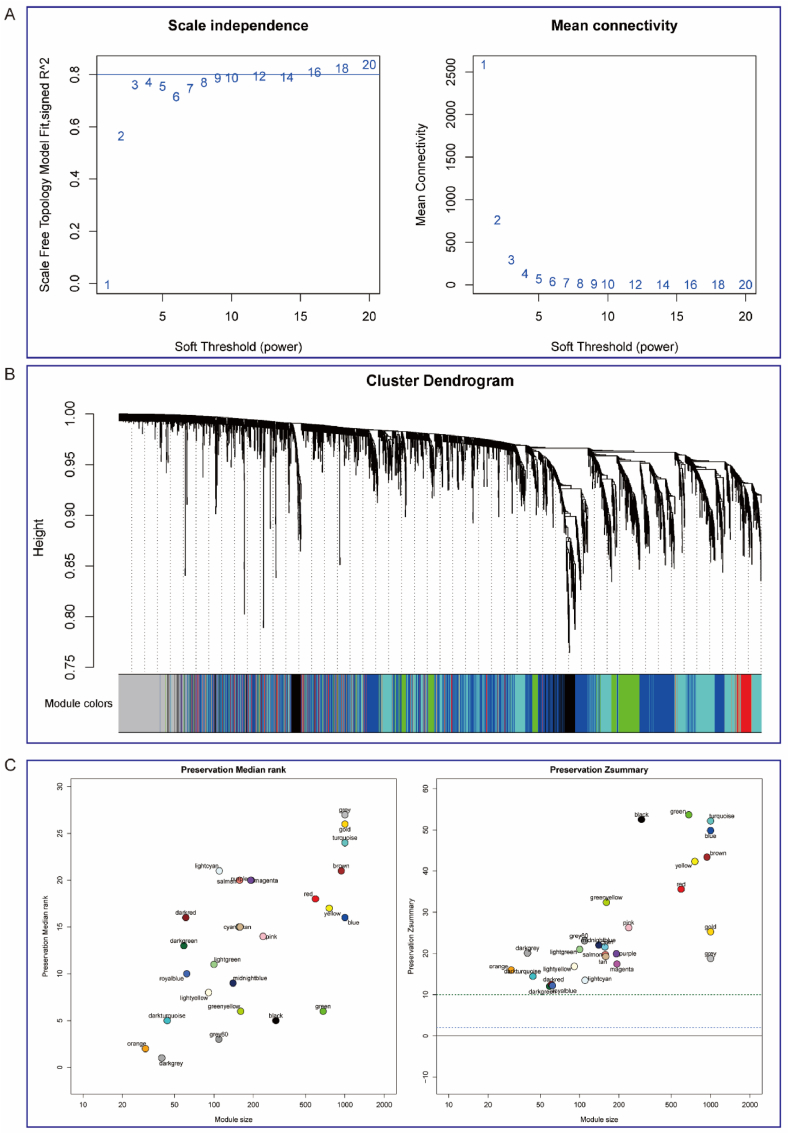
WGCNA analysis. A. The relationship between the scale-free fit index and various soft-thresholding powers, as well as the relationship between average connectivity and various soft-thresholding powers. B. Clustering dendrogram of genes, where different colors represent different modules. C. Module preservation calculated using composite Z-scores. The blue dashed line indicates a Z-score of 2, and the dark green dashed line indicates a Z-score of 10. Modules preserved above this value are considered to have high statistical significance. (For interpretation of the references to color in this figure legend, the reader is referred to the Web version of this article.)

**Fig. 6:**
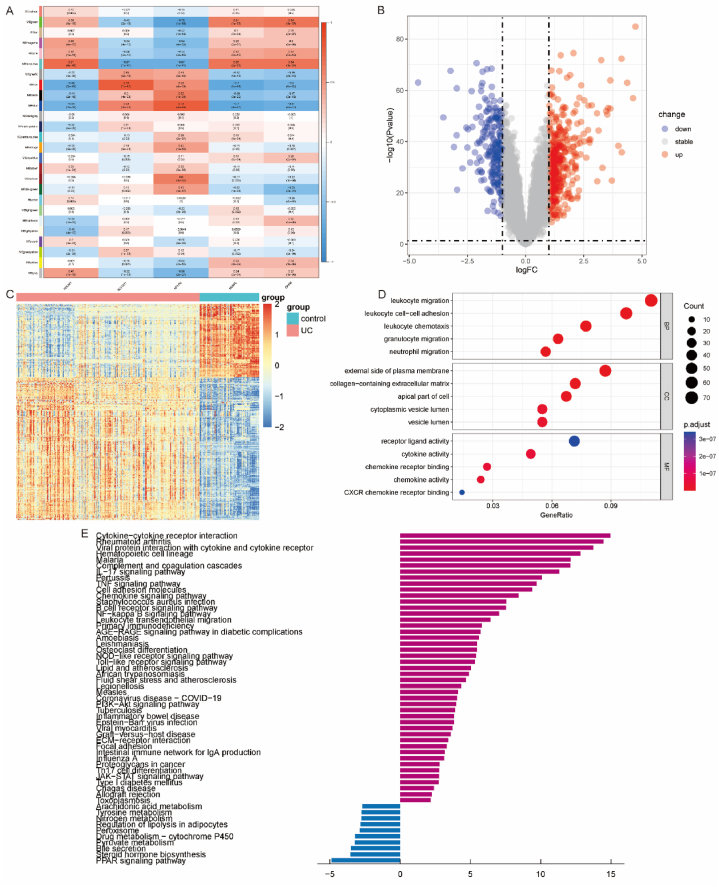
Five immune-related disulfidptosis-associated differentially expressed genes (PDLIM1, SLC7A11, MYH10, NUBPL, OXSM). A. Pearson correlation analysis results between the merged module and immune-related disulfidptosis DEGs. B. Differential analysis of gene modules (6 in total) with Ps ≥ 0.6, visualized using a Volcano plot (|log2 FC| > 1, p < 0.05). C. Heatmap displaying the expression of differentially expressed genes. D. Gene Ontology (GO) analysis. E. Kyoto Encyclopedia of Genes and Genomes (KEGG) functional enrichment analysis.

Due to the close association between disulfidptosis and UC indicated by immune infiltration results, WGCNA was employed to identify genes co-expressed with the target genes. A soft-threshold of 8 was selected (based on the scale-free topology criterion with R2 = 0.80) to build a scale-free network. The adjacency matrix was transformed into a TOM (Topological Overlap Matrix) (Fig. 5A), reflecting node similarity via weighted correlations. This process yielded 25 gene modules (Fig. 5B), all with Z-scores exceeding 10 (Fig. 5C). Correlation analysis revealed strong associations (correlation coefficient >0.60) between the green, turquoise, red, black, blue, and darked modules and these 5 genes (Fig. 6A). Differential gene analysis was then performed on genes within these five modules, applying a threshold of |(FC)| > 1 and p < 0.05 (Fig. 6B). This identified 664 differentially expressed genes, comprising 439 upregulated and 225 downregulated genes (Fig. 6C). “GO and KEGG enrichment analysis” was conducted to explore functions and mechanisms associated with UC using these DEGs. Biological processes were predominantly enriched in leukocyte migration, with GO molecular function analysis indicating enrichment in cytokine activity (Fig. 6D). KEGG enrichment analysis of these DEGs showed significant enrichment in inflammation-related pathways (Fig. 6E), consistent with UC’s known pathogenesis.

### 3.3 Build and validate a nomogram for diagnosing UC

**Fig. 7:**
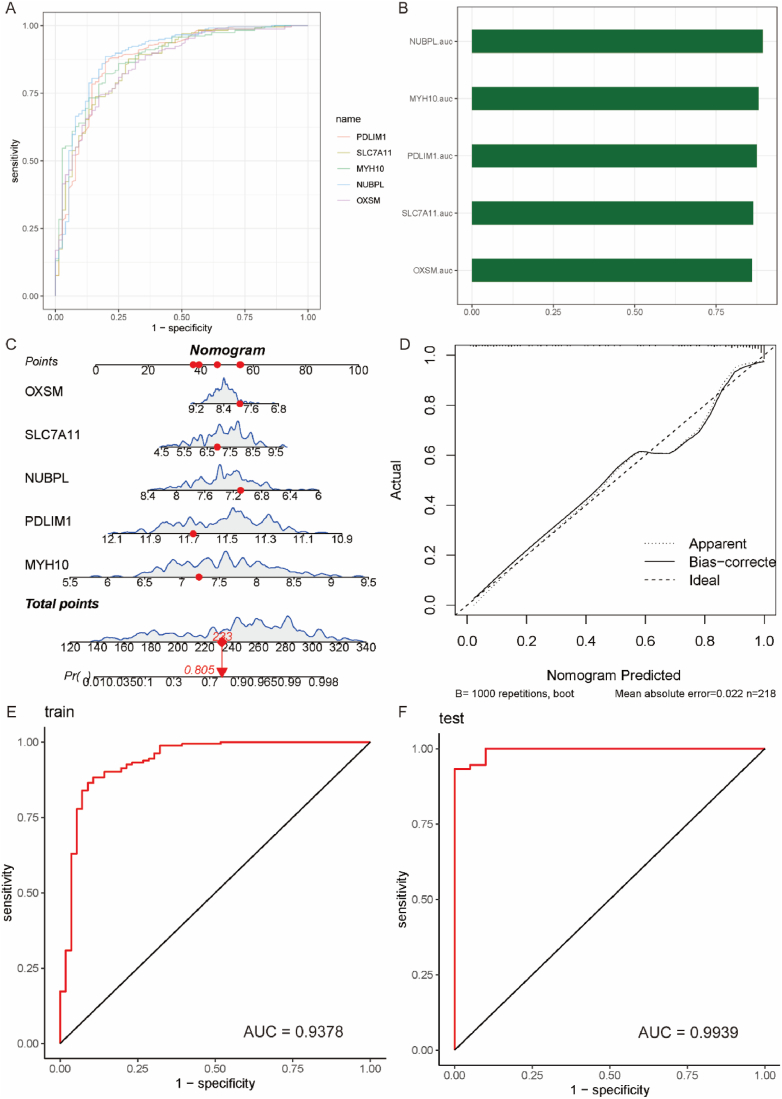
Construction of the Nomogram for diagnosing UC. A. ROC analysis showing the diagnostic capability of the 5 key genes for UC. B. AUC values. C. Constructed Nomogram, with red dots representing randomly selected samples for demonstration. D. Model calibration curve indicating a small deviation between the straight line and the corrected line compared to the ideal line, reflecting good diagnostic consistency and high calibration accuracy. E. Diagnostic capability of the model for UC in the training set (AUC = 0.9378). F. Diagnostic capability of the model for UC in the test set (AUC = 0.9939). (For interpretation of the references to color in this figure legend, the reader is referred to the Web version of this article.)

We employed ROC analysis to evaluate the discriminatory potential of these 5 immune-related disulfidptosis DEGs between UC patients and healthy individuals. The results showed that these genes had good predictive value for diagnosing UC, with AUC values all greater than 0.85 (Fig. 7A and B). To further enhance the ability to diagnose UC, we randomly divided the expression data of these 5 genes into training and validation sets in a 7:3 ratio. In the training set, we constructed a nomogram for diagnosing UC (Fig. 7C). From the generated model calibration curve, it can be observed that the straight line and the post-calibration line deviate minimally from the ideal line, indicating good diagnostic consistency and high calibration accuracy (Fig. 7D). The c-index of the nomogram in the training and validation sets was 0.9378 and 0.9939, respectively (Fig. 7E and F).

Differential Expression and Predictive Role of 5 Disulfidptosis Genes in UC Patients' Intestinal Mucosa Pre- and Post-Biologic Therapy.

The GSE73661 dataset includes gene expression data from UC patients' intestinal mucosa collected before and after treatment with VDZ and IFX. It consists of 12 individuals in the healthy control group, 23 individuals treated with IFX, and 37 individuals treated with VDZ.

**Fig. 8:**
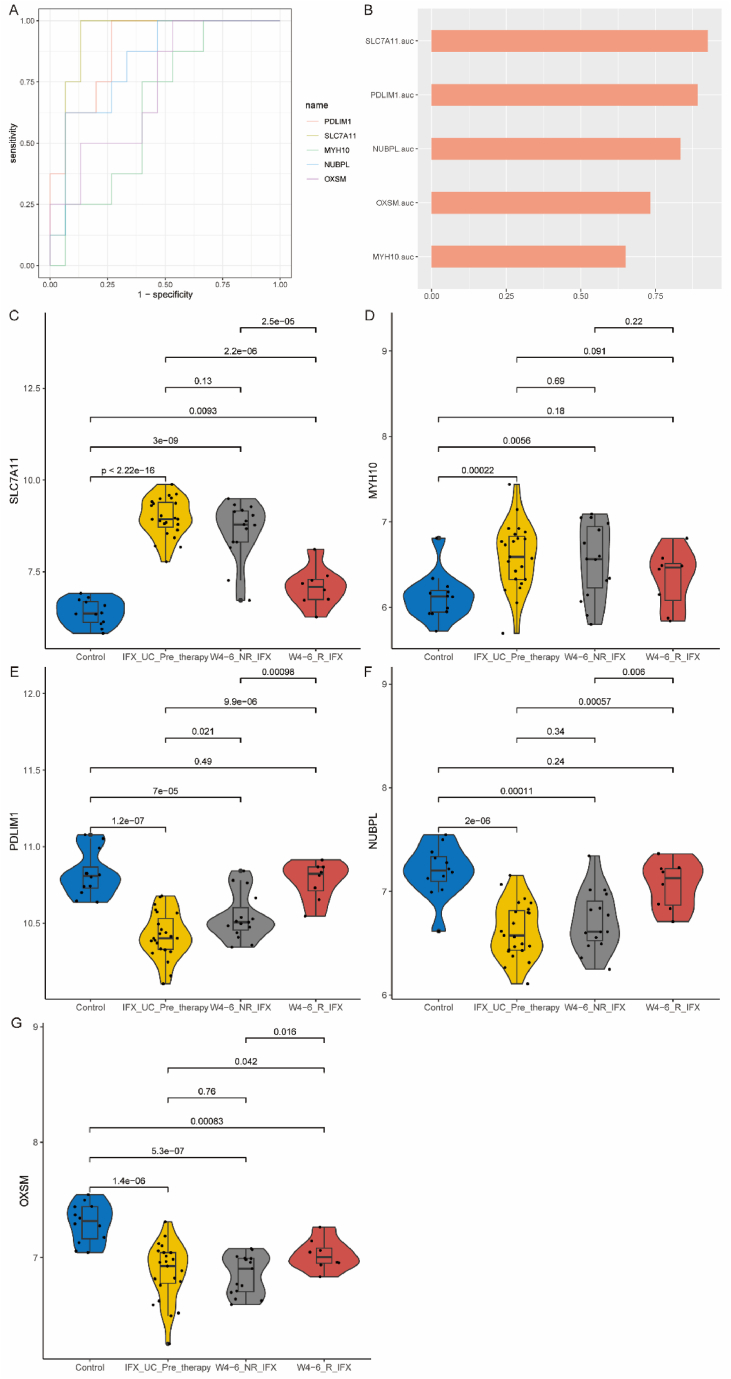
Expression changes of these 5 key genes in UC patients before and after IFX treatment. A. ROC analysis demonstrating the ability of these genes to diagnose treatment effectiveness. B. AUC values. C–G: Changes in the expression of these genes before and after treatment.

In UC patients undergoing IFX treatment, ROC analysis showed that these 5 genes had good predictive ability for the effectiveness of biologic therapy, with AUC values exceeding 0.70 for all genes except MYH10 (Fig. 8A and B). Consistent with previous results, SLC7A11 and MYH10 were upregulated in these UC patients, while the other three genes were downregulated (Fig. 8C–G). In non-responsive patients after IFX treatment, the expression of PDLIM1 was partially upregulated, and the expression of the other 4 genes remained unchanged compared to pre-treatment. In responsive patients after IFX treatment, except for SLC7A11 and OXSM genes, the expression of the other 3 genes was downregulated compared to pre-treatment, with no significant difference in expression compared to the normal control group.

**Fig. 9:**
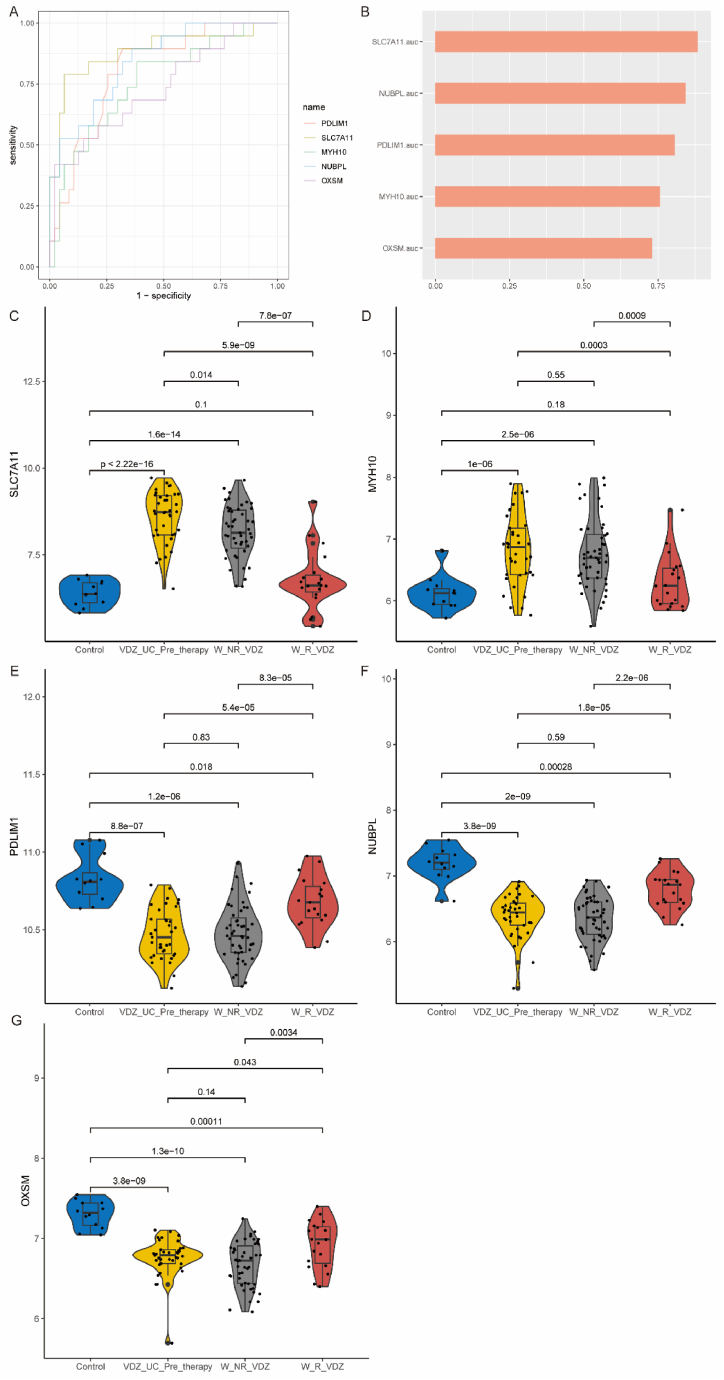
Expression Changes of the five Key Genes in UC Patients Before and After VDZ Treatment. A. ROC analysis demonstrating the ability of these genes to diagnose treatment effectiveness. B. AUC values. C–G: Expression changes of these genes before and after treatment.

Similarly, in UC patients undergoing VDZ treatment, ROC analysis showed that these 5 genes had good predictive ability for the effectiveness of biologic therapy, with AUC values exceeding 0.70 for all genes (Fig. 9A and B). The expression differences of these 5 genes in these UC patients were consistent with previous findings (Fig. 9C–G). In non-responsive patients after VDZ treatment, except for a partial decrease in SLC7A11 expression, the expression of the other 4 genes remained unchanged compared to pre-treatment. In responsive patients after VDZ treatment, the expression of SLC7A11 and MYH10 decreased to normal levels, while the expression of the other three downregulated genes increased but did not reach normal levels.

**Fig. 10:**
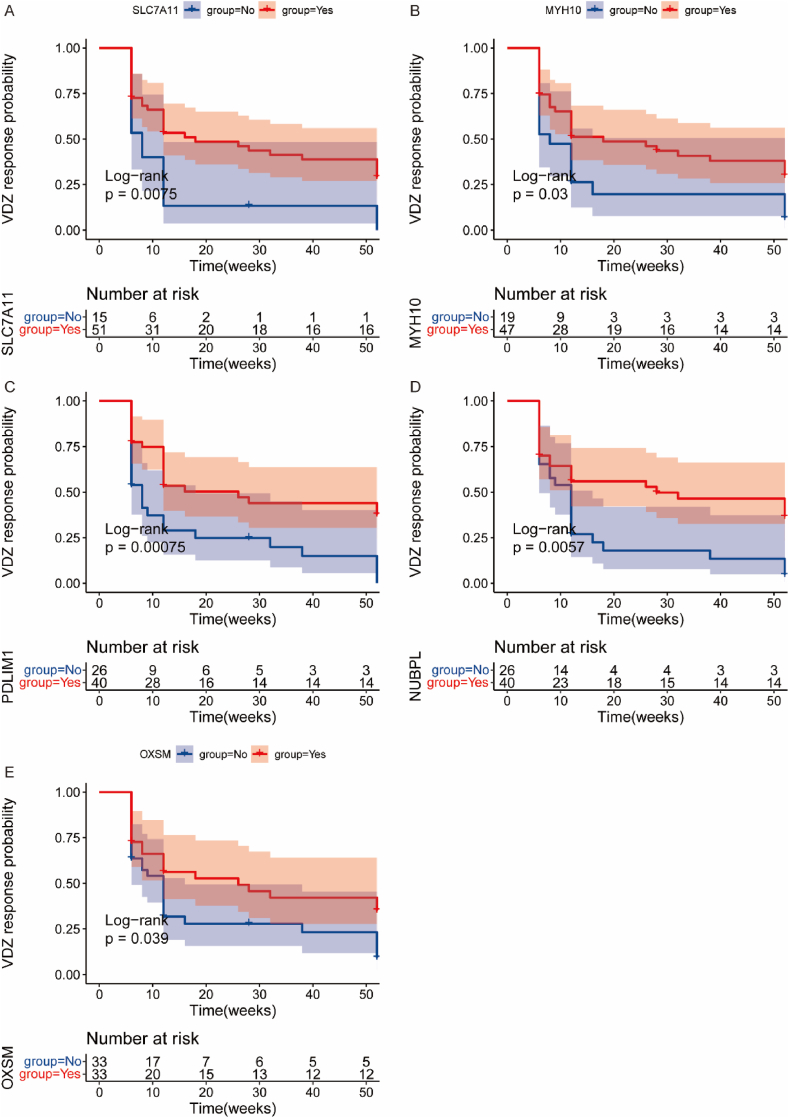
Kaplan-Meier Analysis of the Expression Changes of the Five Core Genes Over Time to Predict the Probability of Effective Response After VDZ Treatment. In the survival analysis, the outcomes were defined as disease remission, classified as “Yes” if the post-treatment expression of upregulated genes in the expression matrix was lower than the average level before treatment or if the post-treatment expression of downregulated genes was higher than the average level before treatment; otherwise, it was classified as “No.” The shaded area represents the 95 % confidence interval.

Given the limited data and absence of specific follow-up time data for patients undergoing IFX treatment, we focused exclusively on patients treated with VDZ for survival analysis. Survival analysis defined results as disease remission, where downregulated genes post-treatment were higher than pre-treatment averages or upregulated genes were lower (Yes), otherwise No. KM analysis demonstrated a time-dependent decrease in the expression of these 5 genes associated with disease remission after VDZ treatment (Fig. 10A–E).

**Fig. 11:**
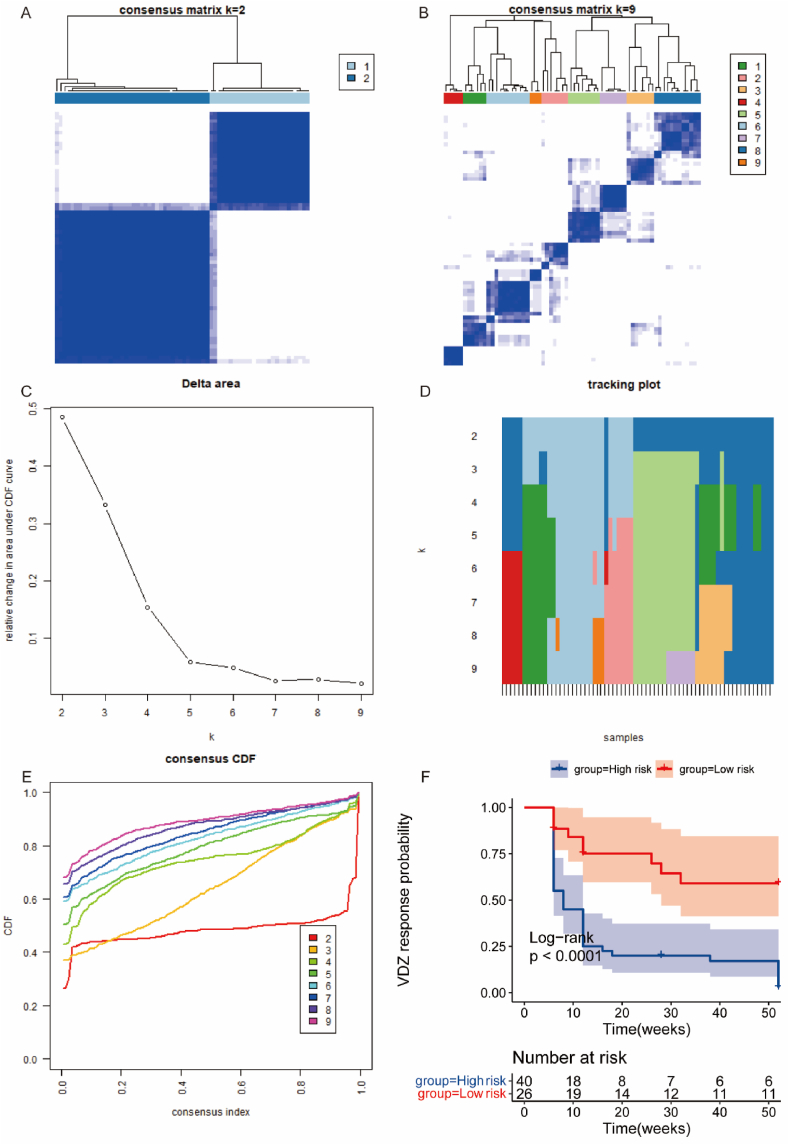
ConsensusCluster Analysis Results. A. Clustering matrix heatmap (K = 2). B. Clustering matrix heatmap (K = 9). C. Delta Area Plot. D. Tracking Plot. E. Cumulative Distribution Function (CDF) plot, indicating that the clustering effect is optimal when K = 2. F. Classification of all patients into high-risk and low-risk groups, where patients in the low-risk group exhibit a significantly higher response rate to VDZ biological treatment than those in the high-risk group.

To further explore the indicative role of disulfidptosis in VDZ treatment, we used the “ConsensusClusterPlus” package to cluster patients receiving VDZ treatment based on the expression of key genes. The results showed that patients could be divided into 2 classes (Fig. 11A–E), representing high-risk and low-risk groups. KM-plot results suggested that over time, patients in the high-risk group had a significantly lower response rate to VDZ biologic therapy than those in the low-risk group (Fig. 11F).

### 3.4 Validating the expression of target genes in UC cell models and clinical tissue specimens

**Fig. 12:**
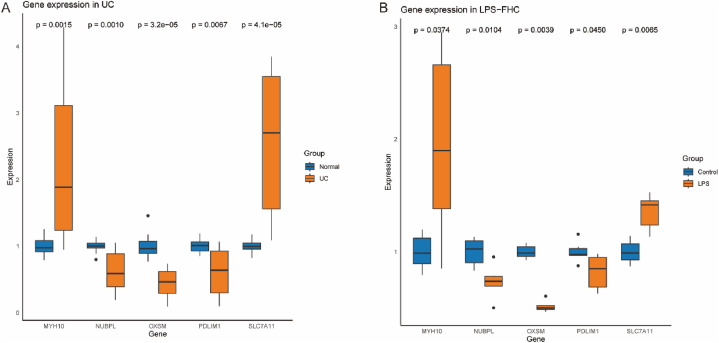
Validating the expression of these 5 target genes in the LPS-induced cell inflammation model and UC intestinal mucosal tissues, all experiments were repeated three times. A: mRNA expression levels of these 5 target genes in 4 UC tissues. B: mRNA expression levels of these 5 target genes in FHC cells induced with 50 ng/ml LPS for 12 h.

To further explore the correlation between the target genes and UC, we obtained multiple points of lesions and relatively normal mucosal tissues from ex vivo intestinal specimens of 4 UC patients who underwent surgery. qPCR results showed consistent expression patterns of these 5 target genes as described previously (Fig. 12 A). Additionally, we further validated the expression of target genes in the LPS-induced cell inflammation model, with qPCR results consistent with those observed in UC tissues (Fig. 12 B).

## 4 Discussion

The intestinal tissue of UC is heterogeneous, comprising different epithelial cells, stromal cells, and immune cells. Dysregulation of the immune system in the intestine can result in recurrent episodes of intestinal inflammation [19]. Dysregulation of the immune response in UC involves both innate and acquired immunity, with interactions between various immune cells leading to excessive local and systemic inflammatory responses [20,21]. The regulation of cell apoptosis and other forms of abnormal cell death plays a crucial role in the pathophysiological processes of various autoimmune diseases, including UC. Despite recent discoveries in disulfidptosis, its role in the intestinal immune response and inflammation in UC remains poorly understood.

In this study, we obtained and analyzed datasets pertaining to Ulcerative Colitis. Through a combination of differential analysis and Lasso regression, we identified seven pivotal genes associated with disulfidptosis. Employing two algorithms, CIBERSORT and ssGSEA, we thoroughly investigated the immune infiltration landscape within the UC intestine. Our focus included examining the correlation between these differentially expressed disulfidptosis genes and various immune cells. Based on the strength of correlation, we selected five key genes (PDLIM1, SLC7A11, MYH10, NUBPL, OXSM). We developed a Nomogram for diagnosing UC utilizing these five genes and rigorously validated its diagnostic efficacy and clinical applicability. Furthermore, by examining their expression levels, we assessed their predictive ability for biologic therapy responses, specifically IFX or VDZ. Lastly, utilizing the expression patterns of these key genes, we stratified patients into two groups, revealing significant differences in disease remission following VDZ treatment. This finding further supports the critical role of disulfidptosis in the progression of UC.

The 24 disulfidptosis-related genes selected for this study were sourced from recent publications. In our differential analysis, 20 of these genes exhibited distinct expression, underscoring a significant correlation between disulfidptosis and UC. Following meticulous screening, we identified 5 key disulfidptosis genes specifically linked to the UC intestine, comprising 2 upregulated genes (SLC7A11, MYH10) and 3 downregulated genes (PDLIM1, NUBPL, OXSM). SLC7A11, a transmembrane protein, facilitates the transport of extracellular cysteine into cells for cysteine and glutathione biosynthesis [22]. In the context of disulfidptosis, heightened SLC7A11 expression expedites cytoplasmic NADPH depletion during glucose starvation, culminating in the accumulation of unreduced disulfides. This process triggers disulfide stress, ultimately leading to disulfide toxicity [8]. Intriguingly, iron death is excessively activated in UC [23], and the elevated expression of SLC7A11 in UC acts as an inhibitor of iron death [24]. Thus, the interplay or synergy of SLC7A11 in iron death and disulfidptosis warrants further exploration. MYH10 emerges as a novel regulator of lipid synthesis and adipocyte function, linked to insulin-dependent glucose transporter translocation [25]. PDLIM1, a scaffold protein, exerts inhibition on the epithelial-mesenchymal transition (EMT) and migration potential of colorectal cancer cells by stabilizing β-catenin at cell-cell junctions [26]. NUBPL, a nucleotide-binding protein-like factor, plays a crucial role as an assembly factor in human mitochondrial complex I, which is the largest component of the mitochondrial respiratory chain [27]. Like MYH10, NUBPL triggers epithelial-mesenchymal transition (EMT), characterized by the reduction in epithelial markers such as E-cadherin and the increase in mesenchymal markers like N-cadherin and vimentin [28]. Mitochondrial 3-oxoacyl-ACP synthase (OXSM) influences cell metabolism and is associated with the proliferation and migration of tumors [29]. Mitochondrial dysfunction can play a role in UC progression by modulating antigen presentation and immune cell homeostasis [30].

The diagnosis of UC patients necessitates a comprehensive approach, incorporating clinical history, endoscopy, pathology, imaging, etc. However, there remains a possibility of misdiagnosis or missed diagnosis, leading to a delay in optimal treatment for patients [1,3]. The Nomogram developed in this study exhibits commendable predictive capabilities and holds significant clinical application value in diagnosing UC. It serves as a guiding tool for IBD physicians in clinical decision-making. The utilization of biologics in the clinical treatment of UC is increasingly prevalent, with agents like VDZ and IFX demonstrating efficacy in a majority of moderate to severe UC patients [31]. Nevertheless, biologics come with a hefty price tag, and the responsiveness of patients to these drugs remains uncertain before usage. Hence, we endeavored to employ these 5 disulfidptosis genes to predict the response to biologic therapy. Intriguingly, our research findings indicate that, prior to treatment, the expression disparities of these 5 pivotal genes in UC, as observed in the GSE73661 dataset, align with previous analytical results. Following biologic therapy, the modulation of upregulated genes or downregulated genes effectively predicts disease remission, showcasing a robust correlation. Conversely, in UC patients exhibiting no response post-treatment, the expression alterations of these 5 genes are inconspicuous.

While our disulfidptosis-related features exhibit excellent capabilities in identifying the immune landscape and diagnosing UC patients, it is crucial to acknowledge certain limitations, for which appropriate methods should be sought in subsequent studies. Owing to individual differences and sequencing batch effects, data analysis relying on public databases may introduce biases in predictive results when compared to real-world scenarios. Despite implementing measures to mitigate this situation, more real-world data is imperative to validate the model’s practicality and the accuracy of immune therapy predictions. Moreover, further prospective studies and foundational research are warranted to fine-tune the specifics of this study.

To summarize, this study utilized bioinformatics analysis and machine learning techniques to identify and analyze immune features related to disulfidptosis across multiple UC datasets. It validated these expression changes in UC patients' intestinal mucosa data from biologic therapy datasets. These findings strongly implicate disulfidptosis in UC progression, advancing our understanding of cell death’s role in UC development and offering new avenues for exploring therapeutic targets in UC.

## Ethical approval

This study has been approved by the Ethics Committee of the Second Xiangya Hospital of Central South University (Approval No: 2022-155). Furthermore, our research strictly adheres to international and national ethical guidelines for research involving human participants, including but not limited to the Declaration of Helsinki. Written informed consent was obtained from all participants. Prior to data collection, we thoroughly explained the purpose of the study, the procedures, potential risks and benefits, and measures to protect their privacy to the participants. All participants explicitly expressed their understanding and voluntarily agreed to participate in the study.

## Funding

This work was supported by the 10.13039/501100001809National Natural Science Foundation of China (82270590).

## Availability of data and materials

The datasets supporting the conclusions of this article are available in the Gene Expression Omnibus (GEO) repository, under accession numbers GSE107499, GSE87466, GSE59071, and GSE73661. These datasets can be accessed through the following links: [GEO Accession viewer (nih.gov)], [GEO Accession viewer (nih.gov)], [GEO Accession viewer (nih.gov)], [GEO Accession viewer (nih.gov)]. Further datasets used and/or analyzed during the current study are available from the corresponding author on reasonable request.

## Data availability statement

The findings of this study are supported by data available in the GEO database, specifically from publicly accessible resources: GSE107499, GSE87466, GSE59071, and GSE73661. In addition, data will be made available on request.

## CRediT authorship contribution statement

**Lichao Yang:** Writing – original draft, Software, Formal analysis, Data curation, Conceptualization. **Lianwen Yuan:** Writing – review & editing, Validation, Supervision. **Ganglei Liu:** Writing – review & editing, Supervision, Methodology, Conceptualization.

## Declaration of competing interest

The authors declare that they have no known competing financial interests or personal relationships that could have appeared to influence the work reported in this paper.

## Figures and Tables

**figs1:**